# Defining the susceptibility of colorectal cancers to BH3-mimetic compounds

**DOI:** 10.1038/s41419-020-02815-0

**Published:** 2020-09-10

**Authors:** Ming-Jie Luo, Michelle Palmieri, Chris D. Riffkin, Anuratha Sakthianandeswaren, Tirta Mario Djajawi, Yumiko Hirokawa, Victoria Shuttleworth, David H. Segal, Christine A. White, Duong Nhu, Guillaume Lessene, Margaret Lee, Peter Gibbs, David C. S. Huang, Oliver M. Sieber, Jia-nan Gong

**Affiliations:** 1 The Walter and Eliza Hall Institute of Medical Research, Melbourne, VIC Australia; 2 School of Medicine, Tsinghua University, Beijing, China; 3 Departments of Medical Biology, University of Melbourne, Melbourne, VIC Australia; 4 Department of Pharmacology and Therapeutics, University of Melbourne, Melbourne, VIC Australia; 5 Department of Medicine (Melbourne Medical School - Western Precinct), University of Melbourne, Melbourne, VIC Australia; 6 Department of Medical Oncology, Western Health, Melbourne, VIC Australia; 7 Department of Medical Oncology, Eastern Health, Melbourne, VIC Australia; 8 Department of Surgery, University of Melbourne, Melbourne, VIC Australia; 9 Department of Biochemistry and Molecular Biology, Monash University, Melbourne, VIC Australia; 10 Present Address: Institute of Cellular Medicine, Newcastle University Medical School, Newcastle upon Tyne, UK

**Keywords:** Cancer therapy, Drug development

## Abstract

Novel targets are required to improve the outcomes for patients with colorectal cancers. In this regard, the selective inhibitor of the pro-survival protein BCL2, venetoclax, has proven highly effective in several hematological malignancies. In addition to BCL2, potent and highly selective small molecule inhibitors of its relatives, BCLxL and MCL1, are now available, prompting us to investigate the susceptibility of colorectal cancers to the inhibition of one or more of these pro-survival proteins. While targeting BCLxL, but not BCL2 or MCL1, on its own had some impact, most (15/17) of the immortalized colorectal cancer cell lines studied were efficiently killed by the combined targeting of BCLxL and MCL1. Importantly, these in vitro findings were confirmed in a xenograft model and, interestingly, in all (5/5) patient derived tumor organoids evaluated. Our results lend strong support to the notion that BCLxL and MCL1 are highly promising targets for further evaluation in efforts to improve the treatment of colorectal cancers.

The direct induction of apoptosis by the BCL2-selective inhibitor, venetoclax (ABT-199), has led to significant progress in treating several blood cancers^1^. Whether BCL2, or its close relatives such as BCLxL or MCL1, is critical for maintaining the survival of solid cancers, including colorectal cancers (CRCs), is not fully defined. We addressed this question by testing immortalized human CRC cell lines in vitro and in vivo, importantly undertaking additional tests of the efficacy of such BH3-mimetic drugs in patient-derived tumor organoids since these are most likely to inform us of the potential clinical utility of such drugs^2^.

Previous studies have implicated BCLxL and MCL1 in maintaining the survival of CRCs^3–5^, but their relative importance has not been clearly established. Thus, we independently tested a panel of 17 well-characterized human CRC cell lines with small molecules^6^ that selectively and potently inhibit BCL2 (venetoclax), BCLxL (A1331852), or MCL1 (S64845). Targeting just BCLxL could induce killing in 3/17 lines, but inhibiting just MCL1 or BCL2 proved ineffective in any of the cell lines tested even after prolonged drug treatment (Fig. 1a, Supplementary Fig. 1).

**Fig. 1:**
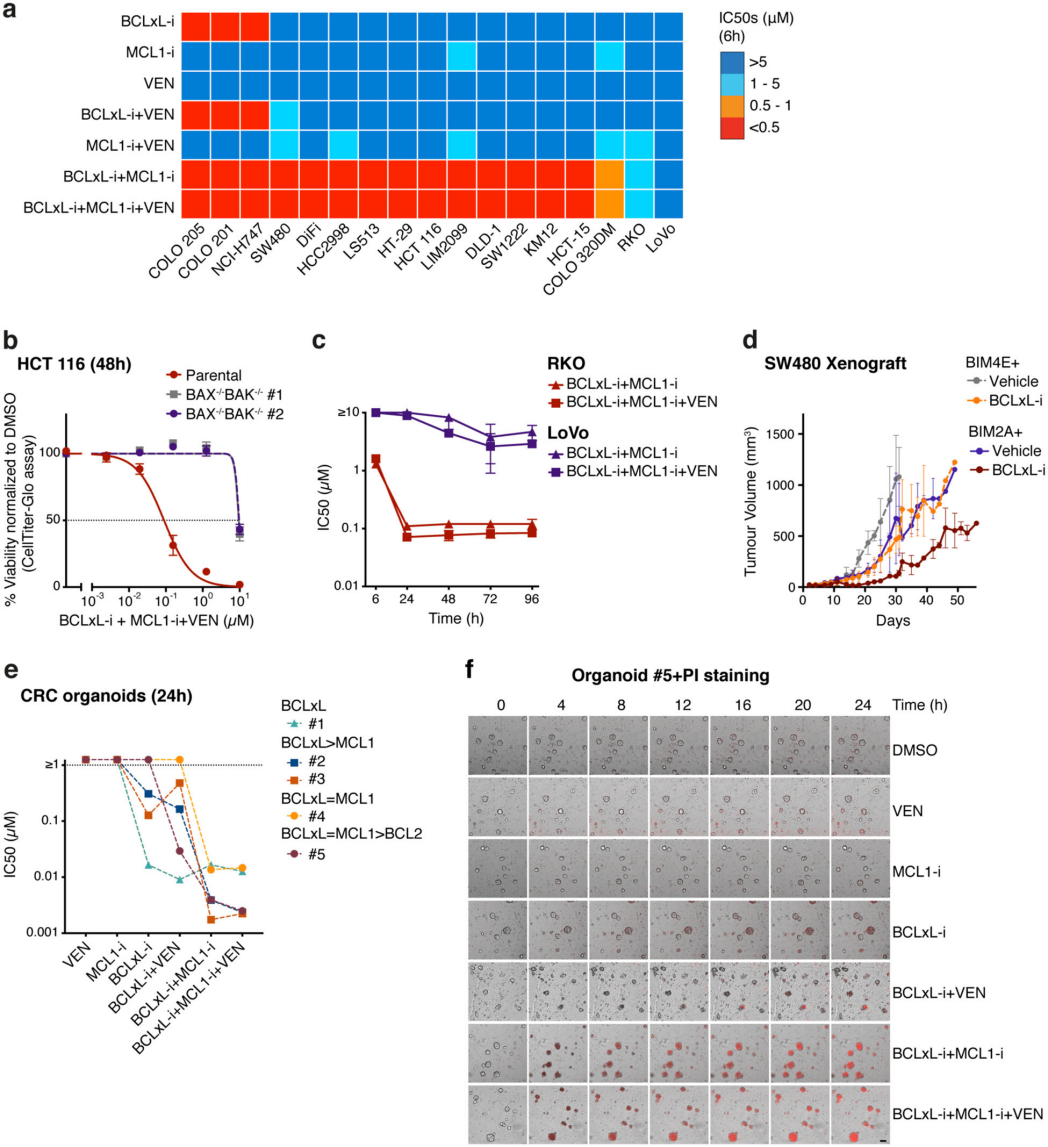
Colorectal cancer cells are highly susceptible to the dual inhibition of BCLxL and MCL1. **a** In vitro sensitivity of a panel of colorectal cancer cell lines to the BH3-mimetic drugs. Seventeen CRC lines were treated with 0–10 μM of BH3 mimetics targeting BCLxL, MCL1, and BCL2, alone or in equimolar combinations (1:1 or 1:1:1). Cell viability was determined after 6 h. A discrete heatmap representation of the mean IC_50_s is shown; red represents potent killing (IC_50_ < 0.5 μM), whereas blue indicates refractoriness (IC_50_ > 5 μM). See Supplementary Table 1 for detailed information of the cell lines used. **b** Killing caused by the combined action of inhibitors targeting BCLxL, MCL1, and BCL2 is mediated by BAX and BAK. The viability of parental HCT 116 cells or a *BAX*^−/−^*BAK*^−/−^ HCT 116 sub-clone was determined 48 h after the combined inhibition of BCLxL, MCL1, and BCL2. **c** LoVo cells are highly refractory to apoptosis induction. Sensitivity (mean IC_50_s ± SD) of RKO or LoVo cells treated with the indicated combinations of BH3 mimetics for 6–96 h is shown. **d** Expression of the MCL1-selective peptide^10^, BIM2A, enhances the in vivo suppression of tumor growth by the BCLxL inhibitor. The SW480 cells engineered to inducibly express BIM2A or BIM4E (inert control) were inoculated subcutaneously into immunodeficient NSG (NOD SCID IL-2Rγ^−/−^) mice and treatment commenced 1 week later with 25 mg/kg of the BCLxL inhibitor A1331852 on weekdays for 2 weeks together with doxycycline-containing food to induce expression of BIM2A or BIM4E. Tumor sizes were monitored every 2–3 days and the data shown represent the mean tumor volumes ± SD of three mice in each group. **e** Sensitivity of patient-derived tumor organoids to the BH3 mimetics. Patient-derived tumor organoids (*n* = 5) were treated with 0–1.25 μM of the indicated BH3-mimetic, alone, or in equimolar combinations (1:1 or 1:1:1). Cell viability was determined 24 h later and the mean IC_50_s of two independent experiments are shown. The relative dependency of each organoid on BCLxL, MCL1, and/or BCL2 is inferred. **f** Rapid induction of apoptosis by BH3-mimetic treatment. The viability of organoid #5 treated with different BH3 mimetics (see **e**) was determined by PI (red) staining and imaged at indicated time points (see Supplementary Video 1). Scale bar = 100 µm. Cell viability in **a**–**c**, **e** was determined using CellTiter-Glo assays; data in **b**, **c** represent the means ± SD from three independent experiments. See Methods in Supplementary Material for experimental details.

This result, suggesting the primacy of BCLxL in CRC, prompted us to ask if MCL1 or BCL2 played secondary roles. Strikingly, 15/17 lines were susceptible when BCLxL and MCL1 were both targeted and co-inhibiting them killed as efficiently as combined treatment with all three BH3 mimetics. Thus, while BCLxL is the primary survival factor in CRC, MCL1 must play a secondary role and BCL2 is relatively unimportant. Of note, the killing induced by these BH3 mimetics was clearly through the specific induction of apoptosis it was fully abrogated in the absence of the apoptosis effectors BAX/BAK (Fig. 1b). Two of 17 lines (RKO and LoVo) remained viable even after 6 h exposure to the triple drug combination. RKO cells showed delayed responses (Fig. 1c), but in contrast, the LoVo cells were highly refractory even with prolonged treatment. In this regard, it is known that the LoVo cell line harbors inactivating mutations in *BAX*^7^. We then sought to confirm the importance of BCLxL and MCL1 in vivo, by testing mice engrafted with the CRC cell line SW480. While modest suppression of tumor growth could be seen by targeting just BCLxL or MCL1, a deeper impact was observed by co-targeting both of these pro-survival proteins (Fig. 1d).

Next, we tested whether this conclusion holds in patient-derived tumor organoids^2^. Remarkably, 3/5 tumor organoids (CRC#1–3) were variably sensitive to BCLxL inhibition alone (Fig. 1e). CRC#1 relies predominantly on BCLxL, while MCL1 was also important in CRC#2–4, and BCL2 contributed additionally in CRC#5. Notably, marked killing of all five was achieved by combined inhibition of BCLxL, MCL1, and BCL2 (Fig. 1e and Supplementary Fig. 2a). The rapid killing of these organoids by these BH3 mimetics was confirmed by the killing kinetics with propidium iodide staining analyzed by microscopic imaging (Fig. 1f, Supplementary Fig. 2b, and Supplementary Video 1).

Our study and others^3–5,8^ pinpoint the importance of BCLxL and MCL1 for maintaining the survival of CRC cells. While the co-targeting of BCLxL and/or MCL1 may well be challenging because of on-target toxicities^9^, the action of the BH3 mimetics could be restricted to tumor cells with antibody–drug conjugates to preferentially target tumor tissues. Our findings will also be useful to develop rational combinations with standard-of-care agents, for example, combining BCLxL inhibition with cytotoxic agents that degrade MCL1, or, more generally, to evaluate BH3-mimetic therapy as sensitizers for agents used to treat CRC. Our study highlights the importance of further laboratory studies to investigate the full potential of BH3-mimetic drugs for treating CRCs, particularly for the large unmet need of patients who are fit for further therapies, but are refractory to all current standard-of-care treatment approaches.

## Supplementary information

Supplementary Materials

Supplementary video
