# Supplementary material for: Defining the susceptibility of colorectal cancers to BH3-mimetic compounds
Source: Cell Death Dis. 2020 Sep 10;11(9):735. doi: 10.1038/s41419-020-02815-0 (PMC7483443; doi:10.1038/s41419-020-02815-0)
Supplement: Supplementary file 1 — Supplementary Materials [file 41419_2020_2815_MOESM1_ESM.pdf]

**Supplementary Materials**

The Supplementary Materials include:

1. Supplementary Methods
2. Supplementary Table
3. Supplementary Figures and Legends
4. Supplementary Video
5. Supplementary References

## Supplementary Methods

**Cell lines** Details of all cell lines used in this study are provided in Supplementary Table 1. The *BAX*$^{-/-}$ *BAK*$^{-/-}$ HCT 116 cells were described previously[1]. The colorectal cancer (CRC) lines and the human embryonic kidney cell line HEK293T were cultured in DMEM supplemented with 10% fetal bovine serum (FBS) at 37°C in 10% $CO_2$. The human leukemic cell lines (RS4;11, MV4-11, DOHH2) and myeloma cell line (KMS-12-PE) were cultured in RPMI-1640 supplemented with 10% FBS at 37°C in 5% $CO_2$. The myeloma cell line AMO1 was cultured in RPMI-1640 medium supplemented with 20% FBS and H929 was cultured in RPMI-1640 supplemented with 10% FBS and 50μM 2-mercaptoethanol. All media contained 1% penicillin and streptomycin. Cell lines were authenticated by short tandem repeat (STR) analyses using the GenePrint 10 System (Promega) at the Australian Genome Research Facility, and confirmed to be mycoplasma free using the Lookout Mycoplasma PCR Detection kit (Sigma-Aldrich, cat #MP0035) or the MycoAlert mycoplasma detection kit (Lonza, cat #LT-07).

**Lentivirus production and infection** Lentiviruses were produced using 3$^{rd}$ generation lentivirus packaging plasmids pMDLg/pRRE (Addgene, cat #12251), pRSV-Rev (Addgene, cat #12253), and pCMV VSV-G (Addgene, cat #12259) transiently transfected into HEK293T cells with the constructs of interest using FuGENE® 6 Transfection Reagent (Promega, cat #2691). The culture media was changed at 16h and supernatants containing infectious viral particles were harvested at 48h and 72h. Supernatant containing virus particles was filtered through a 0.45μM filter. Cells were seeded into 6-well plates at 5 x $10^5$ cells/well. 3mL of culture medium containing viruses was added along with polybrene (Sigma, cat #H9268) to a final concentration of 5μg/mL. Cells were spin-infected (1,800 x g, 25°C, 1h) and then incubated at 37°C for 24h. Cells were then washed and resuspended in fresh culture medium.

**Cell viability assays** To test the response of cancer cell lines to BH3-mimetics, cells were seeded in 96-well plates at 3 x $10^3$ cells/well and treated with serially diluted concentrations of drug (0-10 μM, 5-point 1:8 dilutions) one day later, either alone or in combination (0-10 μM of each drug at 1:1 or 1:1:1 ratio). Compounds used were A1331852 (synthesized by D Nhu and G Lessene, WEHI)[2] to target BCLxL, A1210477 (Active Biochem, cat #A-9036)[3] or S63845 (Active Biochem cat #A-6044)[4] to target MCL1 and venetoclax (ABT-199/GDC-0199/RG-7601; Active Biochem cat #A-1231)[5] to target BCL2. Cell viability was then determined using CellTiter-Glo assay (Promega, cat #G9241) according to the manufacturer's instructions. In some experiments, the cell viability was determined by Annexin V (BD Biosciences cat #560931)/propidium iodide (PI, Sigma cat #P4864) staining and analysed using an LSR-Fortessa flow cytometer (Becton Dickinson). All FACS data were analyzed using FlowJo software. Percentage cell viability was calculated by normalizing to the viability of cells treated with DMSO (vehicle control). GraphPad Prism software was used to calculate the concentration at which cell viability was reduced to 50% (IC50).

***In vivo* xenograft model** 5 x $10^6$ SW480 cells transduced with a doxycycline-inducible vector expressing BIM2A[6] or BIM4E[7] were subcutaneously inoculated into the right flank of female NOD SCID IL-2Rγ$^{-/-}$ (NSG) mice. The inoculation volume (100μL per mouse) comprised a 50:50 mixture of cells in DMEM and Matrigel (Corning, cat #354248). On day 7, mice in the BIM2A or BIM4E group were randomized for treatment with the vehicle (control) or 25 mg/kg BCLxL-i A1331852 on weekdays for two weeks. The formulation of BCLxL-i for oral dosing is 60% Phosal 50 PG, 27.5% PEG-400, 10% ethanol and 2.5% DMSO. The expression of BIM variants was induced with doxycycline administered in food. An electronic calliper was used to measure the dimensions of tumors every 2-3 days. Tumor volume was estimated using the following equation: volume = 1/2 × length × width × width. All animal experiments were

approved by the Walter and Eliza Hall Institute Animal Ethics Committee and conducted according to its guidelines (ethics approval number 2017.007).

**Patient-derived organoids and drug treatment** Organoids used in this study were collected from patients recruited from the Royal Melbourne Hospital, Box Hill and Western Hospitals (all in Melbourne, Australia). Ethics approval was obtained from the Melbourne Health Human Research Ethics Committee (HREC) under HREC Project number 2016.249. Organoids were established as per published protocols[8].

To determine the response of patient-derived organoids to BH3-mimetics, established organoids were resuspended as single cells in DMEM/F12 with Matrigel (Corning, cat #354234) and growth factors as per published protocols[8] and seeded at 4,000 cells/well in 384 optical well plates (Nunc). Cells were cultured for 7 days to enable organoid establishment before drug treatment. BCLxL-i, MCL1-i and venetoclax were prepared as single, double or triple combinations and added to the organoids in a 5-point 10-fold dilution series from 1.25$\mu$M to 0.005$\mu$M with addition of 5$\mu$g/mL PI. Organoids were imaged on the Zeiss Live Cell Axio Observer fluorescent microscope at 5x magnification. An incubation system ensured temperature and $CO_2$ levels remained constant during the course of the experiment. Images were acquired every hour for 24h, with a z-stack of 10 slices at 25$\mu$m intervals. At the experimental endpoint, cell viability was also determined using CellTiter-Glo assay according to the manufacturer's instructions.

**Supplementary Table 1. Cell lines used in this study**

| Cell Line | Cancer Type | Cellosaurus ID | Reference/Commercial Source |
|---|---|---|---|
| COLO 201 | Colorectal cancer | CVCL_1987 | ATCC |
| COLO 205 | Colorectal cancer | CVCL_0218 | ATCC |
| COLO 320DM | Colorectal cancer | CVCL_1989 | ATCC |
| DiFi | Colorectal cancer | CVCL_6895 | Cosmic-CLP |
| DLD-1 | Colorectal cancer | CVCL_0248 | ATCC |
| HCT 116 | Colorectal cancer | CVCL_0291 | ATCC |
| HT-29 | Colorectal cancer | CVCL_0320 | ATCC |
| LIM2099 | Colorectal cancer | CVCL_4436 | Cell Bank Australia |
| LoVo | Colorectal cancer | CVCL_0399 | ATCC |
| LS513 | Colorectal cancer | CVCL_1386 | ATCC |
| NCI-H747 | Colorectal cancer | CVCL_1587 | ATCC |
| RKO | Colorectal cancer | CVCL_0504 | ATCC |
| SW1222 | Colorectal cancer | CVCL_3886 | ECACC |
| HCC2998 | Colorectal cancer | CVCL_1266 | ATCC |
| HCT-15 | Colorectal cancer | CVCL_0292 | ATCC |
| KM12 | Colorectal cancer | CVCL_1331 | ATCC |
| SW480 | Colorectal cancer | CVCL_0546 | ATCC |
| RS4;11 | Acute lymphocytic leukemia | CVCL_0093 | ATCC |
| MV4-11 | Acute myeloid leukemia | CVCL_0064 | ATCC |
| DOHH2 | B cell lymphoma | CVCL_1179 | ATCC |
| AMO1 | Multiple myeloma | CVCL_1806 | DMSZ |
| H929 | Multiple myeloma | CVCL_1600 | ATCC |
| KMS-12-PE | Multiple myeloma | CVCL_1333 | DMSZ |

Fig.S1

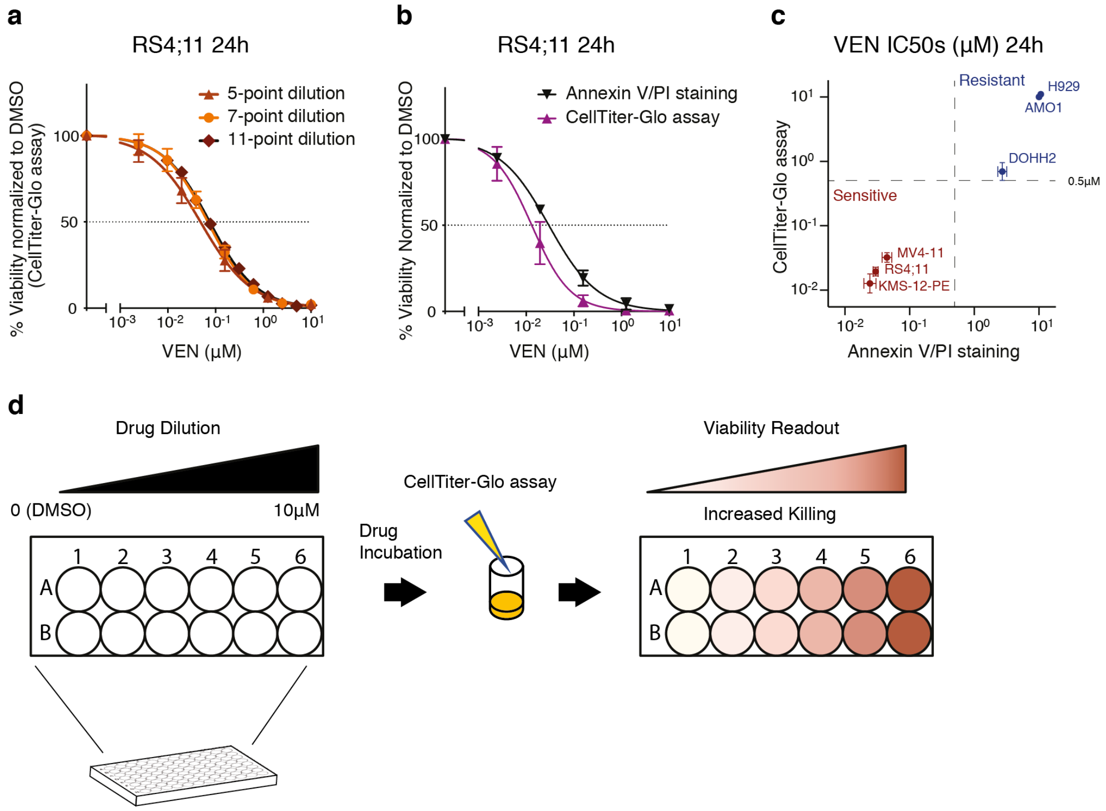


**Supplementary Fig. 1 Optimizing large-scale screening with BH3-mimetic compounds**

(**a**) Impact of varying drug titrations on IC50 values. RS4;11 cells were treated with VEN (0-10µM; 11-point 1:2 dilutions, 7-point 1:4 dilutions, or 5-point 1:8 dilutions starting at 10µM) and the cell viability determined 24h later using CellTiter-Glo assay.

(**b**) Comparison of CellTiter-Glo and Annexin V/PI staining assays to determine cell viability. RS4;11 cells were treated with VEN (0-10µM, 5-point 1:8 titration) and cell viability was determined 24h later using the CellTiter-Glo assay or Annexin V/PI staining followed by flow cytometric analysis.

(**c**) Scatter plot of VEN IC50s in different cell lines determined by two distinct assays. Identical experiments to (**b**) were performed in a panel of human blood cancer cell lines. Some lines were more resistant (IC50>0.5*µ*M) or than others (IC50<0.5*µ*M) to VEN.

(**d**) Summary of platform for screening with BH3-mimetic compounds. Cells were seeded into white opaque 96-well plates at 3,000 cells/well and then treated with BH3-mimetic compounds (0-10µM, 5 point 1:8 titration). Cell viability was determined using CellTiter Glo assay and normalized to that observed with the DMSO controls. GraphPad Prism software was used to calculate the concentration at which cell viability was reduced to 50% (IC50).

Data in (**a-c**) are the means ± SD from 3 independent experiments.

Fig.S2

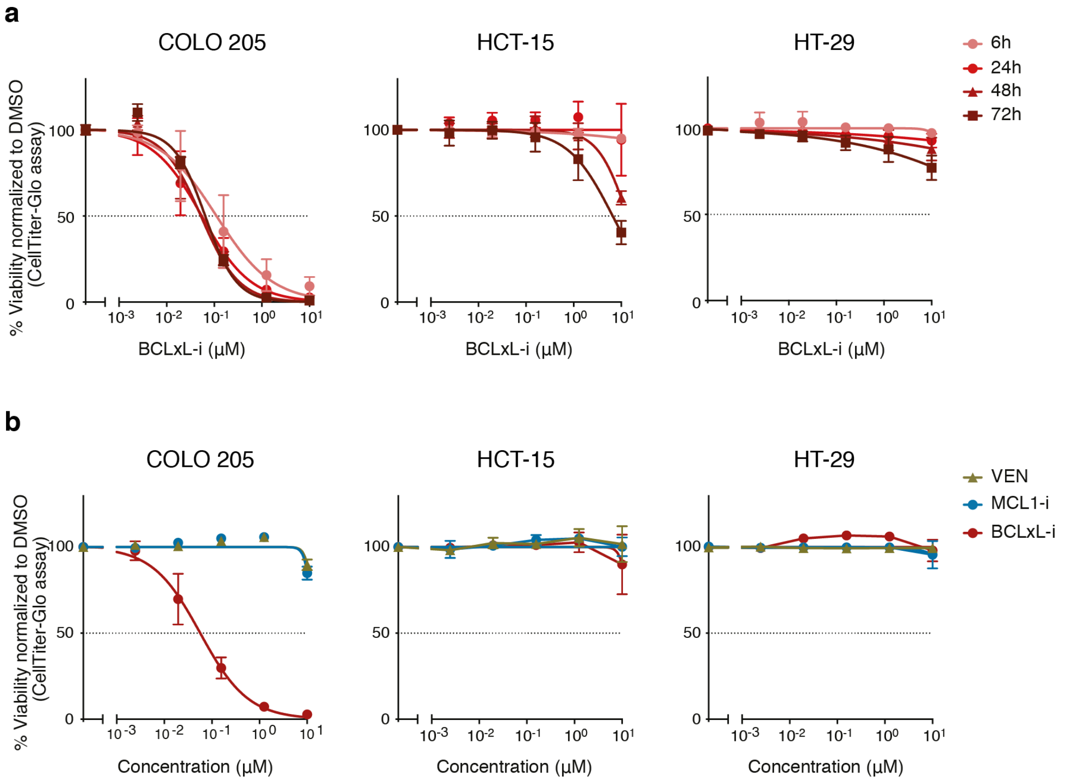


**Supplementary Fig.2 Time course of the responses of colorectal cancer cell lines to BH3-mimetic treatment**

(**a**) Response of indicated colorectal cancer cell lines to BCLxL-i treatment for 6-48h.

(**b**) Response of indicated colorectal cancer cell lines to the BH3-mimetic compounds targeting BCLxL, MCL1 or BCL2 at 24h.

Cell viability was determined using CellTiter-Glo assay. Data in both panels are the means ± SD of 3 independent experiments.

**Fig.S3**

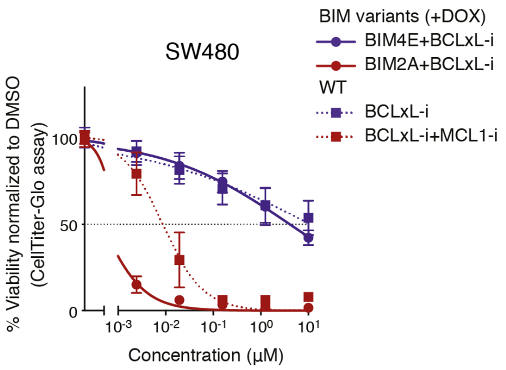


**Supplementary Fig.3 Response of SW480 cells *in vitro* to inhibiting BCLxL and MCL1**

The response of this cell line to inhibition of BCLxL for 24h (in blue) was markedly enhanced by pharmacologically targeting MCL1 with a small molecule inhibitor (red squares, dotted line) or genetically with a selective peptide, BIM2A (red circles, dotted line)[6]. BIM4E served as the inert control[7]. Cell viability was determined using CellTiter-Glo assay; data shown are the means ± SD of 3 independent experiments.

**Fig.S4**

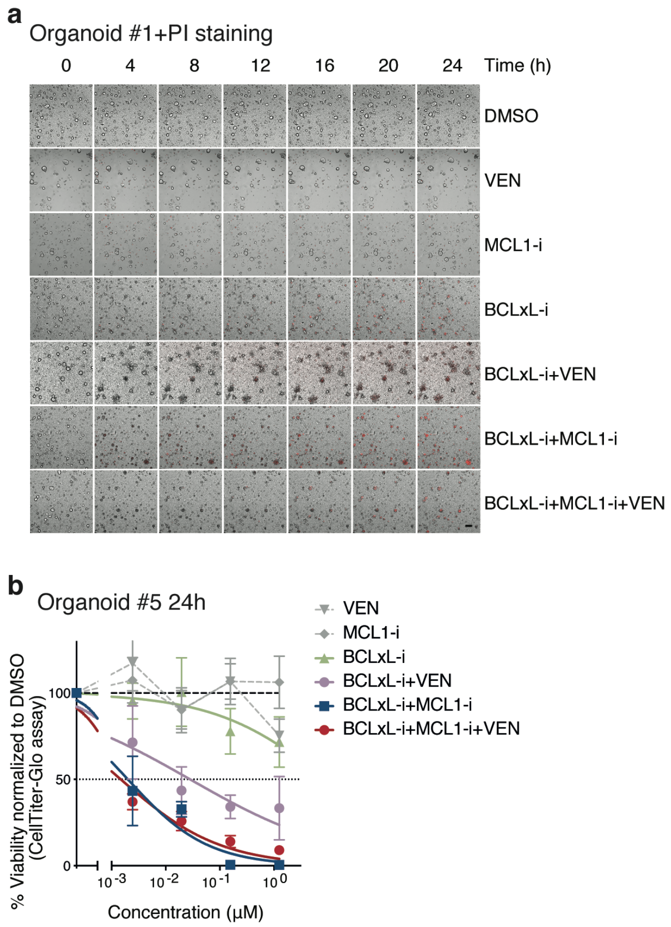


**Supplementary Fig.4 Response of patient-derived organoids to BH3-mimetic treatment.**

(**a**) Patient derived organoid CRC#1 was treated with 0-1.25µM of the indicated BH3-mimetic compound, either alone or in equimolar combinations (1:1 or 1:1:1). Cell viability was determined by PI staining. Images of PI staining at indicated time points are shown. Scale bar=100$\mu$m.

(**b**) The dose response of patient-derived organoid CRC#5 to indicated BH3-mimetic treatment. Data shown are the means ± SD of a representative experiment performed in triplicate.

**Fig.S5**

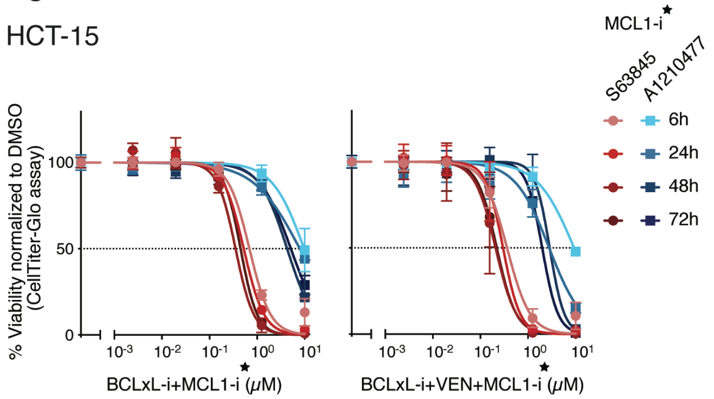


**Supplementary Fig.5 The comparison between two MCL1 inhibitors, S63845 and A1210477.**

HCT-15 cells were treated with the indicated drug combinations with either S63845 (red) or A1210477 (blue) to target MCL1. The cell viability was determined 6-72h later using CellTiter-Glo assay. Data shown are the means ± SD of 3 independent experiments.

**Supplementary Video Response of patient derived CRC tumor organoid to BH3 mimetic compounds** (file labeled "Organoid CRC#5.mp4")

CRC#5 was treated for 24 hours with 1.25μM of the BCLxL inhibitor A1331852 alone, with equimolar addition of the MCL1 inhibitor S63845, or with both the MCL1 inhibitor S63845 and the BCL2 inhibitor venetoclax. The wells were imaged every hour for 24 hours and the samples were stained with PI to detect cells dying by apoptosis. The images are a representative of 3 independent experiments. Scale bar=200μm.

## Supplementary References


1. Wang C, Youle RJ. Predominant requirement of Bax for apoptosis in HCT116 cells is determined by Mcl-1's inhibitory effect on Bak. *Oncogene* 2012, **31**(26)**:** 3177-3189.

2. Leverson JD, Phillips DC, Mitten MJ, Boghaert ER, Diaz D, Tahir SK*, et al.* Exploiting selective BCL-2 family inhibitors to dissect cell survival dependencies and define improved strategies for cancer therapy. *Science translational medicine* 2015, **7**(279)**:** 279ra240.

3. Leverson JD, Zhang H, Chen J, Tahir SK, Phillips DC, Xue J*, et al.* Potent and selective small-molecule MCL-1 inhibitors demonstrate on-target cancer cell killing activity as single agents and in combination with ABT-263 (navitoclax). *Cell Death Dis* 2015, **6**(1)**:** e1590.

4. Kotschy A, Szlavik Z, Murray J, Davidson J, Maragno AL, Le Toumelin-Braizat G*, et al.* The MCL1 inhibitor S63845 is tolerable and effective in diverse cancer models. *Nature* 2016, **538**(7626)**:** 477-482.

5. Souers AJ, Leverson JD, Boghaert ER, Ackler SL, Catron ND, Chen J*, et al.* ABT-199, a potent and selective BCL-2 inhibitor, achieves antitumor activity while sparing platelets. *Nat Med* 2013, **19**(2)**:** 202-208.

6. Lee EF, Czabotar PE, van Delft MF, Michalak EM, Boyle MJ, Willis SN*, et al.* A novel BH3 ligand that selectively targets Mcl-1 reveals that apoptosis can proceed without Mcl-1 degradation. *The Journal of cell biology* 2008, **180**(2)**:** 341-355.

7. Chen L, Willis SN, Wei A, Smith BJ, Fletcher JI, Hinds MG*, et al.* Differential targeting of prosurvival Bcl-2 proteins by their BH3-only ligands allows complementary apoptotic function. *Mol Cell* 2005, **17**(3)**:** 393-403.

8. Sato T, Stange DE, Ferrante M, Vries RG, Van Es JH, Van den Brink S*, et al.* Long-term expansion of epithelial organoids from human colon, adenoma, adenocarcinoma, and Barrett's epithelium. *Gastroenterology* 2011, **141**(5)**:** 1762-1772.
